# Development of Chromatin Regulator-related Molecular Subtypes and a Signature to Predict Prognosis and Immunotherapeutic Response in Head and Neck Squamous Cell Carcinoma

**DOI:** 10.2174/0115680096274798231121053634

**Published:** 2024-01-04

**Authors:** Juntao Huang, Ziqian Xu, Zhenzhen Wang, Chongchang Zhou, Yi Shen

**Affiliations:** 1 Department of Otolaryngology-Head and Neck Surgery, Ningbo Medical Center Lihuili Hospital, The Affiliated Lihuili Hospital of Ningbo University, Ningbo, Zhejiang, China;; 2 Department of Dermatology, Ningbo First Hospital, Ningbo, China;; 3 Centre for Medical Research, Ningbo No.2 Hospital, Ningbo, China;; 4 School of Medicine, Ningbo University, Ningbo, China

**Keywords:** Chromatin regulators, signature, prognosis, immunotherapy, head and neck squamous cell carcinoma, CR-related genes

## Abstract

**Background:**

Chromatin regulators (CRs) serve as indispensable factors in tumor biological processes by influencing tumorigenesis and the immune microenvironment and have been identified in head and neck squamous cell carcinoma (HNSCC). Hence, CR-related genes (CRRGs) are considered potential biomarkers for predicting prognosis and immune infiltration in HNSCC. In this study, we established a novel signature for predicting the prognosis and immunotherapeutic response of HSNCC.

**Methods:**

A total of 870 CRRGs were obtained according to previous studies. Subsequently, patients in the TCGA-HNSC cohort were divided into different clusters based on the expression of prognostic CRRGs. Kaplan‒Meier (K‒M) survival analysis was conducted to compare the prognosis in clusters, and the CIBERSORT and ssGSEA methods assessed the immune infiltration status. In addition, the differences in immunotherapeutic responses were determined based on the TICA database. Furthermore, the differentially expressed CRRGs between clusters were identified, and the predictive signature was established according to the results of univariate Cox, least absolute shrinkage and selection operator regression analysis, and multivariate Cox. The predictive effects of the risk model were evaluated according to the area under the receiver operating characteristic (ROC) curve (AUC) in both the training and external test cohorts. A nomogram was established, and survival comparisons, functional enrichment analyses, and immune infiltration status and clinical treatment assessments were performed. In addition, the hub gene network and related analysis were conducted with the Cytohubba application.

**Results:**

Based on the expression of prognostic CRRGs, patients were divided into two clusters, in which Cluster 1 exhibited a better prognosis, more enriched immune infiltration, and a better immunotherapeutic response but exhibited chemotherapy sensitivity. The AUC values of the 1-, 3- and 5-year ROC curves for the risk model were 0.673, 0.732, and 0.692, respectively, as well as 0.645, 0.608, and 0.623 for the test set. In addition, patients in the low-risk group exhibited more immune cell enrichment and immune function activation, as well as a better immunotherapy response. The hub gene network indicated ACTN2 as the core gene differentially expressed between the two risk groups.

**Conclusion:**

We identified molecular subtypes and established a novel predictive signature based on CRRGs. This effective CRRS system can possibly provide a novel research direction for exploring the correlation between CRs and HNSCC and requires further experimental validation.

## 1 INTRODUCTION

With an increasing number of diagnosed samples (800,000 per year) and deaths (440,000 per year), head and neck squamous cell carcinoma (HNSCC) is considered the most common head and neck cancer, with the top six leading incidences [1-3]. For patients with advanced HNSCC, elimination surgery combined with radiotherapy and chemotherapy is administered as the conventional treatment but is associated with an unsatisfactory prognosis and a low 5-year survival rate of 50% [4, 5]. Individualized treatment is necessary for HNSCC patients as a precise alternative [6]. Recently, surgical resection combined with immunotherapy has improved prognostic results and prolonged overall survival (OS) [7, 8]. Immune checkpoint inhibitor (ICI) therapy, which can activate a patient’s immune defense system, has displayed significant therapeutic effects in identifying and eliminating tumor cells and is considered a novel adjuvant in multiple clinical management strategies [9]. Anti-PD-1/L1 antibodies and CTLA-4 inhibitors are the most representative ICIs and are associated with promising clinical immunotherapeutic responses in patients [10]. However, not all tumor patients obtain ideal results from ICI therapy, and only a minority of patients benefit from immunotherapy. In fact, according to previous studies, the tumor immune microenvironment (TIME), including immune cells, stromal cells, and various cytokines, significantly influences immune escape and weakens the immune response [11]. Several hub genes are involved and participate in the regulation of the TIME, and researchers and doctors can obtain more accurate insight into tumor progression with the assistance of reliable biomarkers and subsequently provide guidance for the treatment of HNSCC. Therefore, it is crucial to develop a novel biomarker to reveal the underlying relationship between the TIME and immunotherapeutic response.

Chromatin regulators (CRs), which are grouped into three categories: DNA methylators, histone modifiers, and chromatin remodelers, serve as indispensable factors in tumor biological processes [12-14]. As indicated in previous studies, CRs can exhibit dysregulated gene expression across tumors, promoting tumorigenesis. This dysregulation occurs at different molecular levels and can be influenced by mutations [15]. Similarly, chromatin structure regulation plays a crucial role in interactions between tumors and immune cells [16]. Tumor cells can escape immune restriction by hijacking this epigenetic mechanism, which can explain the failure of immunotherapy [17]. In addition, pharmaceutical modulation of CRs can normalize impaired immunosurveillance and activate antitumor immune responses [17]. Given these findings, CRs can be considered potential targeted biomarkers that improve the immune response and promote better immunotherapeutic effects. Previous studies have investigated the correlation between HNSCC and CRs from multiple perspectives, including tumor proliferation, invasion, mutation, and metabolism [16, 18]. However, detailed studies exploring the roles of CRs in the survival, immune infiltration microenvironment, immunotherapeutic responses, and chemotherapeutic effects in HNSCC patients are lacking. CRs may have great potential for prognostic prediction and therapeutic guidance, and it is strongly recommended that a novel assessment system based on CRs be established to predict the prognosis of and clinical therapy for HNSCC patients.

Given these findings, we used computational bioinformatics analyses to explore the correlation between CRs and HNSCC and to predict the prognosis, immune landscape, and clinical treatment of HNSCC based on CRs.

## 2 MATERIALS AND METHODS

### 2.1 Data Acquisition and Processing

To obtain an HNSCC cohort, a dataset of HNSCC patients was downloaded from The Cancer Genome Atlas (TCGA) database. The RNA sequencing transcriptome data (downloaded as fragments per kilobase million values), related clinical information (including overall survival (OS) value, survival status, age, sex, tumor grade, clinical stage), and tumor mutation burden (TMB) information were extracted from the TCGA-HNSC dataset, excluding patients with missing OS values. Moreover, the GSE41613 cohort of HNSCC patients was extracted from the Gene Expression Omnibus (GEO) database as an external test dataset. All the above data were processed with R Bioconductor packages *via* R (version 4.1.2) software.

### 2.2 Obtaining the Chromatin Gene Set and Molecular Subtypes

Based on previous studies, 870 CR-related genes (CRRGs) were identified (Table **S1**), and the expression matrix of CRRGs in the TCGA-HNSC dataset was annotated with the utilization of the “limma” R package [13]. Subsequently, concerning the expression of prognostic CRRGs identified by univariate Cox (uni-Cox) proportional hazard regression analysis (*P <* 0.05), patients were regrouped into novel molecular subtypes using the “K-means” method *via* the “ConsensusClusterPlus” R package. Principal component analysis (PCA) was conducted to assess the distribution of CRRGs in clusters, and differences in OS in clusters were compared *via* Kaplan‒Meier (K‒M) survival analysis.

### 2.3 Functional Enrichment Analysis

Gene set variation analysis (GSVA) was conducted to compare the differentially enriched pathways in clusters. With the assistance of the gene set (kegg.v7.4.symbol.gmt), the top 20 differentially enriched signaling pathways were identified and are shown in the heatmap plot according to the criterion of an adjusted *P <* 0.05.

### 2.4 Tumor Microenvironment and Immunotherapeutic Response in Clusters

In addition, the tumor immune microenvironment (TIME) scores were assessed with the estimation of stromal and immune cells in malignant tumor tissues using the expression data (estimate) algorithm. Four kinds of scores, including the immune score, stromal score, estimate score, and tumor purity for each HNSCC patient, were calculated based on the RNA-seq of the TCGA-HNSC cohort. Immune scores and stromal scores were used to predict the level of infiltrating immune and stromal cells, and subsequently, estimate scores and tumor purity were determined based on these scores. Furthermore, the immune cell infiltration status and immune functions were evaluated *via* CIBERSORT and single-sample gene set enrichment analysis (ssGSEA), respectively. Subsequently, a Wilcoxon test was conducted to compare the differences in these evaluating indicators in clusters.

To predict the immunotherapeutic response in different tumor subtypes, the expression of immune checkpoint genes was calculated and compared *via* the Wilcoxon test. In addition, the immunophenoscores (IPSs) were compared to evaluate the efficacy of ICIs, including PD-1 and CTLA4, based on the TCIA database (https://tcia.at/home).

### 2.5 Establishment of the Prognostic Signature and Nomogram

Referring to the criteria of |log2-fold change (logFC)| > 0.585 and false discovery rate (FDR) < 0.05, the differentially expressed CRRGs (DECRRGs) in clusters were selected and illustrated in a heatmap plot. Subsequently, the prognostic DECRRGs identified by the univariate Cox analysis were further selected by least absolute shrinkage and selection operator (LASSO) regression analysis, and a prognostic risk model was constructed with the use of multivariate Cox (multi-Cox) regression analysis. The risk scores were calculated using the following formula: chromatin regulator risk score (CRRS)=∑ coefficient of (DECRRG)^i^ × expression of (DECRRG)^i^. Each patient was assessed with the risk score system and divided into two risk groups (low-risk group and high-risk group) according to the median value of risk scores. The differences in OS were compared by Kaplan‒Meier (K‒M) survival analysis. Additionally, the distribution of risk scores and survival status are shown in a risk plot for both the TCGA and GEO cohorts.

Similarly, the area under the receiver operating characteristic (ROC) curve (AUC) was applied to evaluate the predictive effects of the risk signature. Moreover, concerning the results of univariate and multivariate Cox regeneration, the risk scores and other independent clinical predictive factors were pooled to develop the nomogram for further prediction.

### 2.6 Correlation Analysis between the Risk Score and Clinical Features

To further assess the predictive effects of the CRRS system, the distribution of clinical features in the low- and high-risk groups was reflected in the bar plots and box plots. In addition, OS in different clinical subgroups between the low-risk and high-risk groups was compared by K‒M survival analysis.

### 2.7 Correlation Analysis Between the CRRSs and Tumor Mutation Burden

To explore the correlation between the CRRSs and tumor burden mutation (TMB), the mutation frequency of prognostic CRRGs in the risk model was assessed in a waterfall plot, and the interactive correlation was tested with Pearson correlation analysis. Moreover, the top 20 mutated genes between the low-risk group and the high-risk group were also reflected in waterfall plots with the use of the “maftool” R package. The correlation between the CRRS and TMB was explored and analyzed by Spearman analysis. Furthermore, survival analysis was conducted to assess the effects of the CRRS combined with TMB in HNSCC patients.

### 2.8 Expression of Model Genes at the Cellular Level

To further identify the specific type of immune cells expressing model genes in the TIME, two single-cell RNA datasets (GSE103322 and GSE139324) were analyzed *via* the tumor immune single-cell hub (TISCH, http://tisch.comp-genomics.org/home/) database.

### 2.9 Assessment of the TIME and Immunotherapeutic Response in the Risk Model

Similarly, the differences in TIME scores between the two groups were evaluated and compared based on the immune scores, stromal scores, estimate scores, and tumor purity using the “estimate” R package. Moreover, to further explore the relationship between the CRRS and immune infiltration status, the CIBERSORT algorithm was utilized to assess the infiltration status of immune cells in each HNSCC patient. In addition, single-sample gene set enrichment analysis (ssGSEA) was performed to assess the immune functions of each sample. Correlation analysis was conducted to reflect these results and describe the infiltration status. In addition, the correlation of ICI-related gene expression and the CRRS was calculated using the “limma” R package to identify the differential immune checkpoints and predict the potential efficacy of ICI therapy. Similarly, the immunotherapeutic response in HNSCC patients based on the CCRS system was investigated according to the IPS of each HNSCC patient collected from the TCIA database.

### 2.10 Drug Sensitivity of Chemotherapy Agents

The drug sensitivity of five chemotherapy agents commonly administered to HNSCC patients, including cisplatin, docetaxel, gemcitabine, methotrexate, and paclitaxel, was assessed with the use of the “pRRophetic” R package. The half-maximal inhibitory concentration (IC50) values of these five agents for each HNSCC sample were predicted and calculated. The Wilcoxon test was conducted to compare differences in IC50 values between the clusters and CRRS groups. In addition, the correlation between the CRRS and IC50 values was evaluated based on the Spearman correlation test [19].

### 2.11 Constructing a Protein‒protein Interaction Network and Hub Gene Analysis

Concerning the selective criteria of |logFC| > 0.585 and FDR < 0.05, differentially expressed genes (DEGs) were identified between the low-risk and high-risk groups. These DEGs were then pooled and input into the Search Tool for the Retrieval of Interacting Genes/Proteins (STRING) to construct a protein‒protein interaction (PPI) network diagram, which was visualized *via* Cytoscape (version 3.6.2) software. To identify the hub genes, a critical subnetwork was established using the Cytohubba application, which was utilized for the identification of the hub DEGs. Calculated by the algorithm of maximal clique centrality (MCC), the top 10 DEGs were selected from the network ranking by the MCC scores. Furthermore, based on the results of the hub network, further analysis of the topmost genes was conducted, including differential expression, survival, clinical correlation, and immune-related analyses.

## 3 RESULTS

### 3.1 Identification of Chromatin-related Subtypes

**Fig. (1):**
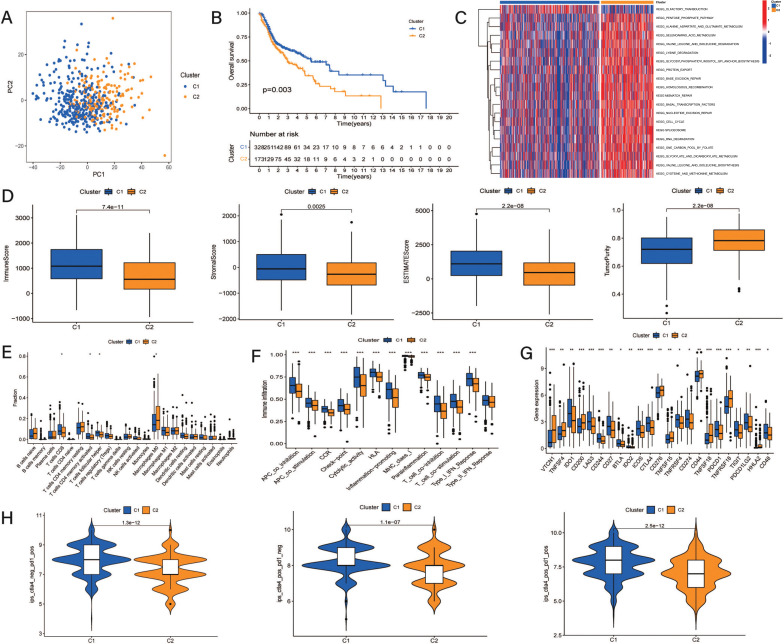
Molecular subtypes based on expression of chromatin regulator related genes. (**A**) Principal component analysis suggested patients can be divided into two clusters clearly; (**B**) K-M survival analysis of OS in clusters; (**C**) GSVA for enriched pathways in clusters; (**D**) Tumor immune environment scores based on the estimate platform; (**E**) Immune cell infiltration according to CIBERSORT; (**F**) Comparison of immune functions between the clusters. (**G**) Comparison about expression of immune checkpoint genes in clusters. (**H**) Immunotherapeutic response based on TCIA database.

After identifying the prognostic CRRGs by uni-Cox analysis, patients were regrouped into two clusters based on the expression of these prognosis-related CRRGs. The PCA suggested that patients in the TCGA-HNSC cohort were distinguished clearly (Fig. **1A**) and that patients in Cluster 1 presented better OS than those in Cluster 2 (Fig. **1B**). In addition, pathways related to protein synthesis, transport, and metabolism, which are considered to play important roles in the biological processes of tumors, were more enriched and active in patients in Cluster 2 (Fig. **1C**).

In addition, as shown in Fig. (**1D**), patients in Cluster 1 had significantly higher immune scores, stromal scores, and estimate scores; nevertheless, the tumor purity in Cluster 1 was much lower. Furthermore, while comparing the infiltration status of immune cells, patients in Cluster 1 exhibited more immune cell infiltration, including CD8^+^ T cells and activated memory CD4 T cells (Fig. **1E**). Similarly, based on the ssGSEA results, Cluster 1 had more activation of immune functions than Cluster 2 (Fig. **1F**). Moreover, comparing the expression of ICI-related genes between the two clusters, Cluster 1 exhibited higher gene expression of CTLA4 and PDCD1, which suggested that patients in Cluster 1 might be more sensitive to CTLA4 and PD-1 inhibitor immunotherapy (Fig. **1G**). The comparative analysis of the TCIA also supported the above results that patients in Cluster 1 exhibited a higher IPS while being treated with CTLA4, PD-1, or a combination of CTLA4 and PD-1 (Fig. **1H**).

### 3.2 Establishment and Validation of a CRRG-related Risk Model

**Fig. (2):**
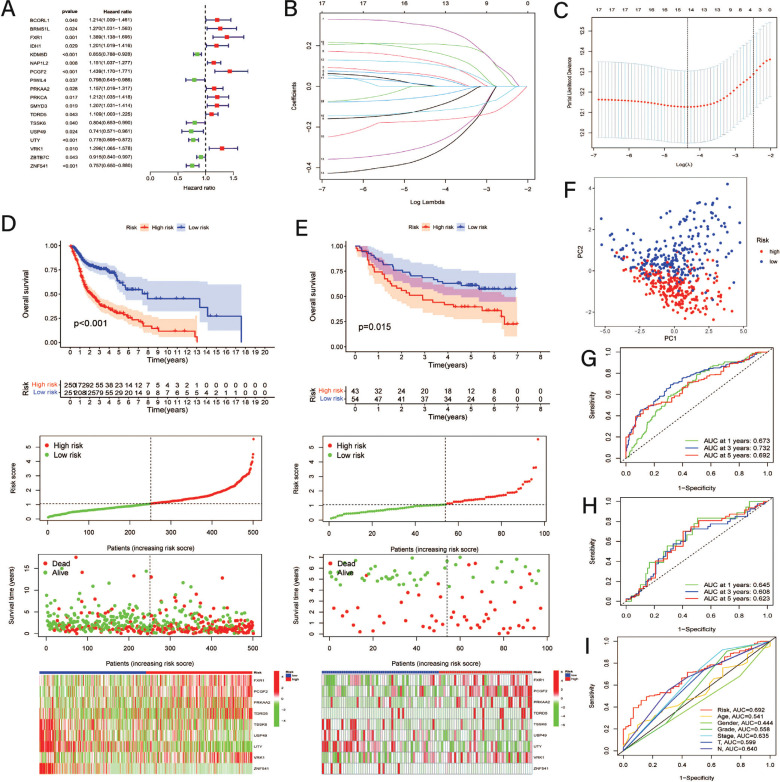
Establishment and validation of a CRRG-related risk model. (**A**) Forest plot about prognostic DECRRGs in clusters; (**B**) Diagram for least absolute shrinkage and selection operator (Lasso) expression coefficients; (**C**) Cross-validation plot for the penalty term of Lasso analysis; (**D**) K-M survival analysis, Exhibition of risk model, Survival time and survival status and Heatmaps of 9 CRRGs expression in TCGA-HNSC cohort; (**E**) K-M survival analysis, Exhibition of risk model, Survival time and survival status and Heatmaps of 9 CRRGs expression in GSE41613 cohort; (**F**) PCA analysis between the risk groups based on model genes; (**G**) 1-, 3-, and 5-year’s AUC values of ROC in TCGA-HNSC cohort; (**H**) 1-, 3-, and 5-year’s AUC values of ROC in GSE41613 cohort; (**I**) Comparison of 5-year’s ROC among risk model and clinical features.

With the criteria of |logFC| > 0.585 and FDR < 0.05, a total of 151 DECRRGs were pooled into uni-Cox proportional hazards, and 18 DECRRGs were identified as prognosis-related genes, as shown in the forest plot (Fig. **2A**). Among them, 11 prognostic CRRGs were considered to increase the risks of HNSCC patients; nevertheless, the remaining 7 CRRGs possibly prolonged OS. Subsequently, based on the results of LASSO and multi-Cox regression analysis (Fig. **2B** and **C**), a novel predictive risk score system was constructed concerning the expression of 9 CRRGs and was divided into a low-CRRS group and a high-CRRS group according to the median value of the CRRSs. The risk score of each HNSCC patient was calculated using the following formula: CRRS= FXR1^exp^ × 0.269282170862417 + PCGF2^exp^ × 0.362681459041864 + PRKAA2^exp^ × 0.151870852873057 + TDRD5^exp^ × 0.130465644507974 - TSSK6^exp^ × 0.39933256377339 - USP49^exp^ × 0.47248492303673 - UTY^exp^ × 0.189418721252206 + VRK1^exp^ × 0.257771725353382 - ZNF541^exp^ × 0.175846447131989. Compared with the differences in OS between the low-CRRS and high-CRRS groups, patients with higher CRRSs presented worse OS than low-CRRS patients in both the TCGA-HNNC and GSE41613 cohorts (Fig. **2D** and **E**). The distinction of the CRRS, survival time, and model-related CRRG expression suggested that this risk model can distinguish HNSCC patients clearly into two groups, which were also determined by PCA (Fig. **2F**). In addition, as indicated by the AUC values of the 1-year, 3-year and 5-year survival ROC curves, this risk model displayed satisfactory predictive effects in both the training (Fig. **2G**) and test (Fig. **2H**) cohorts. Moreover, it also behaved better and more precisely than any other clinicopathological features (Fig. **2I**).

**Fig. (3):**
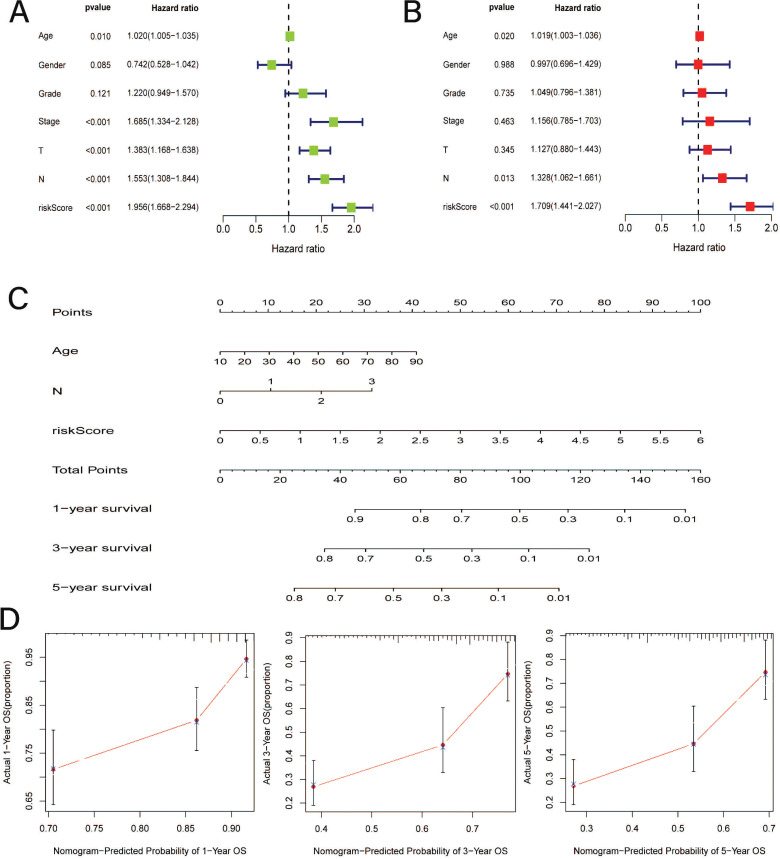
Construction of a predictive nogogram. (**A**) Uni-Cox analyse among clinical features and risk scores. (**B**) Multi-Cox among clinical features and risk scores. (**C**) Establishment of nomogram to predict the 1-, 3-, and 5-year’s prognosis based on ages, stages and risk scores; (**D**) Calibration curves plot.

Similarly, referring to the results of uni-Cox and multi-Cox regression analysis, age, N stage, and the CRRS can be considered independent elements for prognostic prediction, which showed significant differences (*P <* 0.05) both in uni-Cox and multi-Cox forest plots (Fig. **3A** and **B**). Subsequently, a nomogram for further prediction was established (Fig. **3C**), and the high degree of consistency between actual observations and nomogram predictions reflected the satisfactory predictive effects (Fig. **3D**).

### 3.3 Assessment of the CRRS with Different Clinicopathological Features

**Fig. (4):**
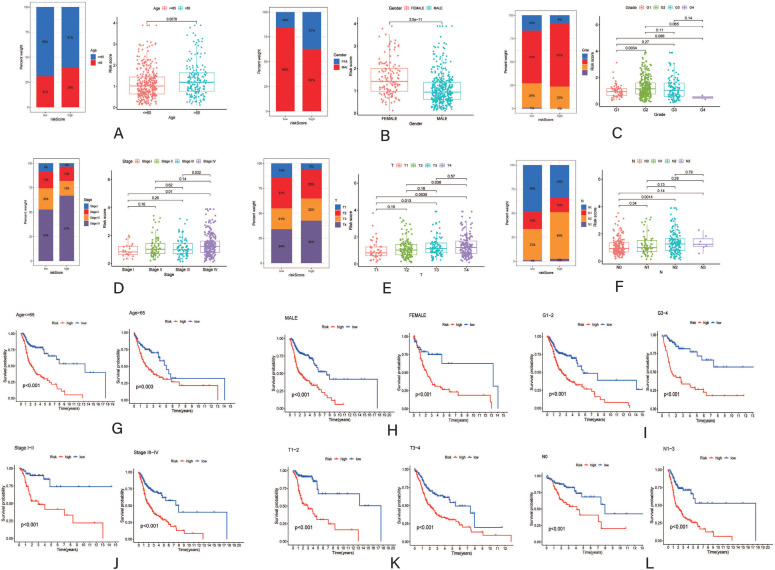
Correlation between CRRSs and clinicopathological features. A-F Distribution and percentage of CRRSs in different clinicopathological features, (**A**) (age), (**B**) (gender), (**C**) (grade), (**D**) (stage), (**E**) (T), (**F**) (N); (**G-L**), Survival analysis between the low-CRRS and high-CRRS groups with different subgroups, (**G**) (age < =65 and >65), (**H**) (male and female), (**I**) (G1-2 and G3-4), (**J**) (Stage I-II and Stage III-IV), (**K**) (T1-2 and T3-4), (**L**) (N0 and N1-3).

To compare the differences and assess the correlation of the CRRS with different clinicopathological features, patients in the TCGA-HNSC cohort were analyzed based on different subgroups. The distinction of patients with different characteristics was reflected in the bar plots. As the boxplots indicated, older male patients appeared to have higher CRRSs than younger female patients, which coincided with previous studies. However, when comparing the CRRS between some subgroups in other clinicopathological features (including grade, stage, T stage, and N stage), there were no significant differences (Fig. **4A**-**F**). Moreover, while conducting subgroup survival analysis with different features, patients with high CRRSs also had worse OS than those in the low-CRRS group (Fig. **4G**-**L**).

### 3.4 Correlation of the Risk Model and TMB

**Fig. (5):**
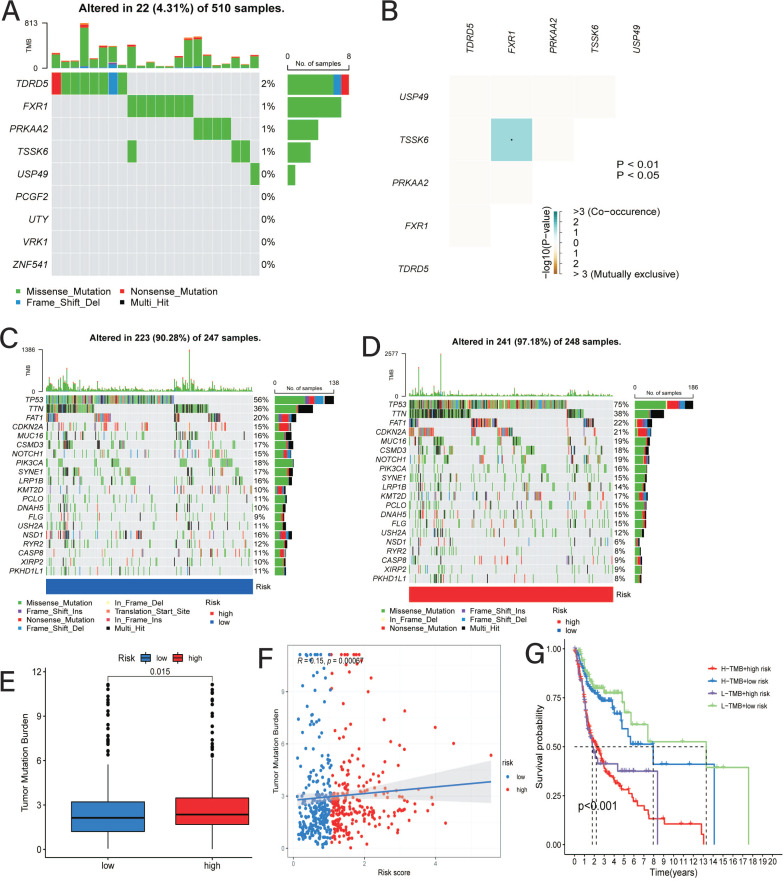
Correlation between CRRS and TMB. (**A**) Mutated frequency of 9 model genes; (**B**) Mutated association among 9 model genes; (**C**) Waterfall plots of topmost 20 mutated genes in the low-CRRS group; (**D**) Waterfall plots of topmost 20 mutated genes in the high-CRRS group; (**E**) Comparison of TMB between the low-CRRS and high-CRRS groups; (**F**) Correlation analysis between CRRS and TMB based on Spearman test; (**G**) Survival analysis for patients combined with TMB and CRRS.

As shown in Fig. (**5A**), the overall mutation rate of nine prognostic genes was low in the TCGA-HNSC cohort, reaching approximately 4.31% in 510 samples. In addition, the correlation plot suggested that the TSSK6 gene was positively associated with the FXR1 gene mutation (Fig. **5B**). Similarly, the mutation rates of the top 20 mutated genes in the TCGA-HNSC cohort were analyzed and displayed in waterfall plots, suggesting that patients in the low-CRRS group exhibited a low TMB frequency (Fig. **5C** and **D**). Moreover, there was a positive correlation between the CRRS and TMB frequency, suggesting that increasing the CRRS may promote tumor gene mutation (Fig. **5E** and **F**). In addition, while performing survival analysis combined with TMB frequency, those with a low CRRS and low TMB had the best prognosis; nevertheless, patients with a high CRRS and high TMB had the poorest prognosis (Fig. **5G**).

### 3.5 Validation of Model Genes *via* TISCH

**Fig. (6):**
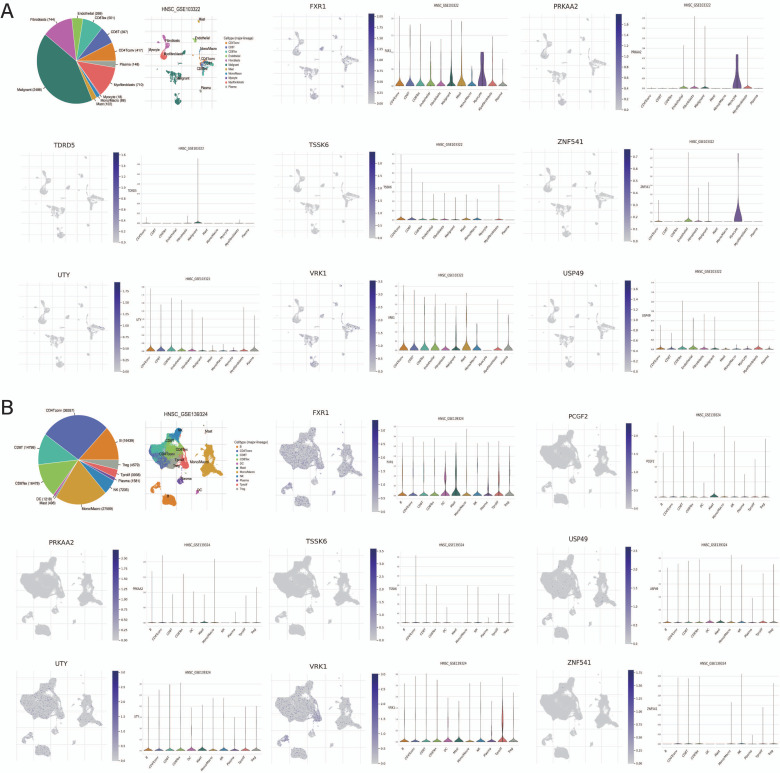
Validation of model genes in single-cell RNA cohorts (GSE103322 and GSE139324) *via* TISCH database. PCGF2 was not detected in GSE103322 as well as TDRD5 was not found in GSE139324.

According to the results of the TISCH database, the expression landscape of model genes among immune cells in the HNSCC TIME reflects 11 different immune cell clusters. As determined by GSE103322 and GSE139324, the FXR1 gene was considered to be expressed at much higher levels than the other seven model genes in the immune system of the TIME. Specifically, referring to the results from GSE103322, FXR1 was mostly expressed in myocyte cells (Fig. **6**).

### 3.6 Functional and Immune-related Analysis Based on the CRRS System

**Fig. (7):**
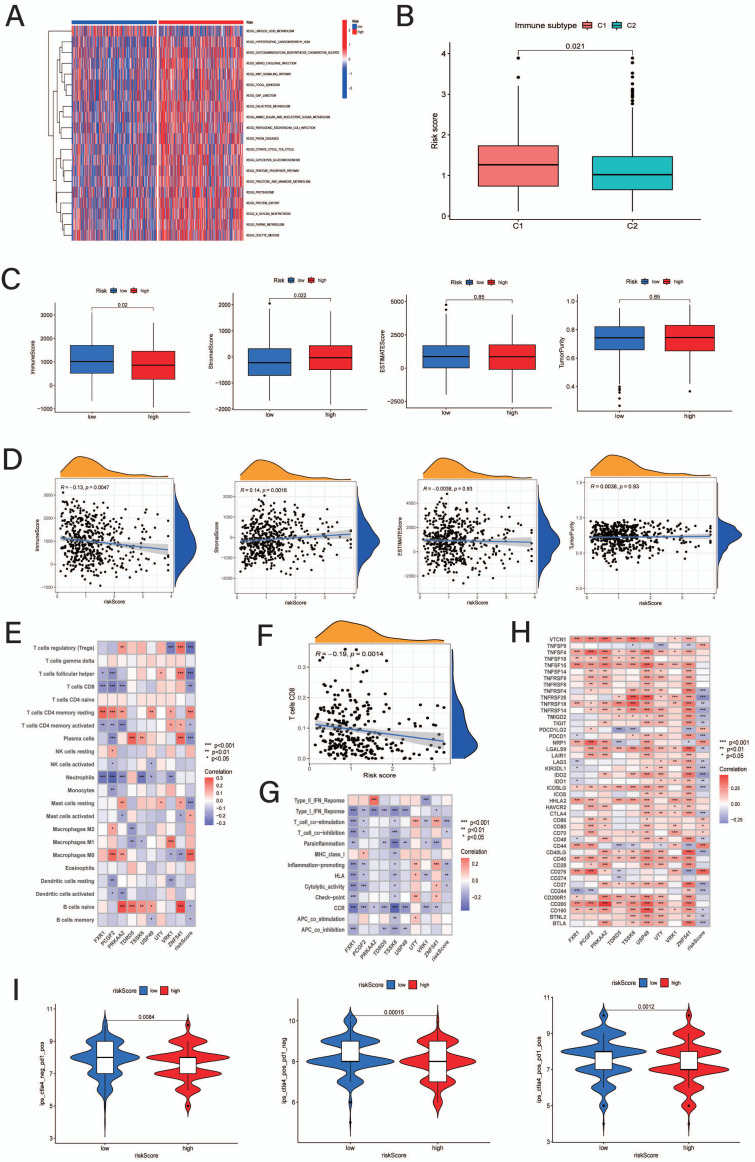
Functional enrichment and immune landscape analysis in CRRS groups. (**A**) Comparative of GSVA about enriched pathways in two CRRS groups; (**B**) Distribution of CRRS in immune subtypes (C1 and C2); (**C**) Comparison of TME score between the low-CRRS and high-CRRS groups; (**D**) Correlation analysis of CRRS with immune scores, stromal scores, ESTIMATE scores and tumor purity; (**E**) Correlation analysis about CRRS, model genes and immune cells based on CIBERSORT; (**F**) Correlation between CRRS and CD8^+^ T cell; (**G**) Correlation analysis about CRRS, model genes and immune functions *via* ssGSEA; (**H**) Correlation analysis about CRRS, model genes and immune checkpoint genes; (**I**) Comparison of IPS between the low-CRRS and high-CRRS groups.

According to GVSA pathway analysis, patients in the low-CRRS group had a more activated linoleic acid metabolism pathway; however, the high-CRRS group had a more enriched pathway than the low-CRRS group, including glycosaminoglycan biosynthesis, chondroitin sulfate, and galactose metabolism (Fig. **7A**). Subsequently, when comparing the CRRSs between the two immune subtypes (C1 and C2), patients in immune Cluster 1 showed higher CRRSs than those in immune Cluster 2 (Fig. **7B**). Moreover, concerning the TIME scores based on the estimate platform, patients in the low-CRRS group had higher immune scores and lower stromal scores; however, there were no significant differences in the estimate scores or tumor purity between the two risk groups (Fig. **7C**). The correlation analysis also supported the Wilcoxon comparison that the CRRS was negatively correlated with immune scores and positively associated with stromal scores (Fig. **7D**). Similarly, as recommended by the correlation plots, the CRRS was negatively correlated with Treg cells, CD8 T cells, follicular helper T cells, activated CD4 T cells, plasma cells, resting mast cells, naive B cells and memory B cells but positively associated with resting memory CD4 T cells, active mast cells and M0 macrophages (Fig. **7E**). These correlations with TIME indicated that patients with a high CRRS may have had an immunosuppressive status and that those with lower scores possibly had more active immune activation. The correlation rate of CD8 T cells was -0.19, indicating that increasing the CRRS may decrease the immunotherapeutic response to PD-1 inhibitor therapy (Fig. **7F**). Similarly, patients with low CRRSs exhibited more activation of immune functions according to the ssGSEA results, which also supports the above results (Fig. **7G**). In addition, the prognostic model CRRGs were positively associated with most ICI-related genes; nevertheless, the CRRSs exhibited a negative correlation with the ICI genes CTLA4, PDCD1, and PDCD1LG2 (Fig. **7H**). Therefore, patients with higher CRRSs may display more highly expressed ICI genes but are not sensitive to PD-1- or CTLA4-inhibiting agents. Moreover, the comparison of IPS also supports these results that the low-CRRS group behaved more sensitively to immunotherapy with PD-1 and CTLA4 inhibitors (Fig. **7I**).

### 3.7 Assessment of Chemotherapy

**Fig. (8):**
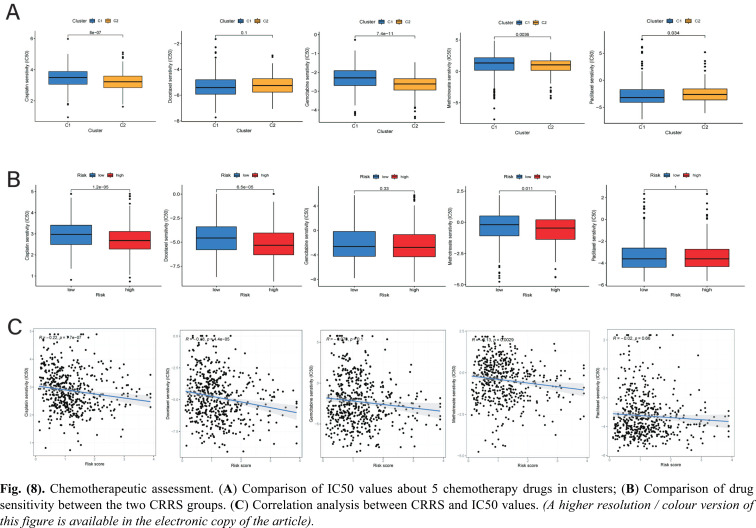
Chemotherapeutic assessment. (**A**) Comparison of IC50 values about 5 chemotherapy drugs in clusters; (**B**) Comparison of drug sensitivity between the two CRRS groups. (**C**) Correlation analysis between CRRS and IC50 values.

With the “pRRophetic” packages, the chemotherapeutic responses to cisplatin, docetaxel, gemcitabine, methotrexate, and paclitaxel for each HNSCC patient were assessed according to the IC50 value. The Wilcoxon comparison between the two chromatin subtypes suggested that patients in Cluster 1 were more sensitive to paclitaxel; however, Cluster 2 exhibited lower IC50 values for cisplatin, gemcitabine, and methotrexate. Additionally, there were no differences in docetaxel between the two clusters (Fig. **8A**).

Similarly, while comparing the drug sensitivity between the two CRRS groups, the CRRSs were negatively correlated with the IC50 values of cisplatin, docetaxel, and methotrexate, suggesting that patients with higher CRRSs exhibited more sensitivity to these chemotherapy agents. That is, patients in the high-CRRS group possibly had a better chemotherapeutic response than those in the low-CRRS group. Otherwise, there were no significant differences between the low-CRRS and high-CRRS groups when comparing IC50 values for gemcitabine or paclitaxel (Fig. **8B** and **C**).

### 3.8 Construction of the PPI Network and Assessment of Hub Genes

**Fig. (9):**
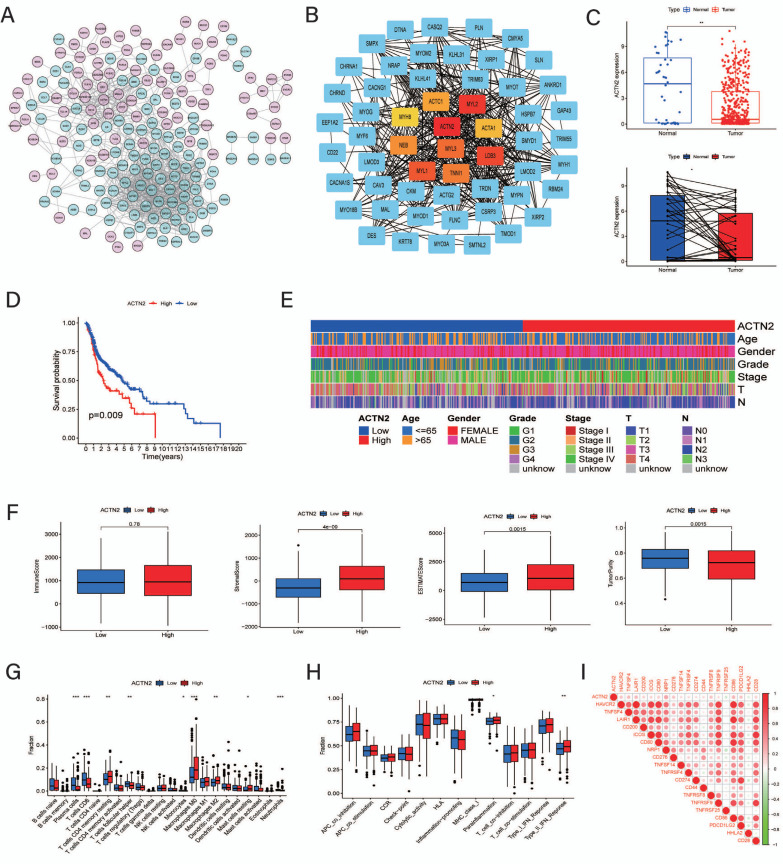
Protein‒protein interaction network and hub gene analyses. (**A**) Protein‒protein interaction of differentially expressed genes between the two CRRS groups, the pink cycle suggested this gene highly expressed in the high-CRRS groups whereas the bright blue cycle indicated highly expressed in the low-CRRS groups; (**B**) Sub-network about hub genes based on Cytohubba; (**C**) Comparing expression of ACTN2 between normal and HNSCC samples; (**D**) Survival analysis between the low-ACTN2 and high-ACTN2 groups; (**E**) Heatmap of relationship among ACTN2 and clinical features; (**F**) TME scores in two ACTN2 groups; (**G**) Immune cell infiltration with different expression of ACTN2; (**H**) Comparison of immune functions between the low-ACTN2 and high-ACTN2 groups; (**I**) Expression correlation among ACTN2 and immune checkpoint genes.

According to the criteria |logFC| > 0.585 and FDR < 0.05, a total of 424 DEGs were identified as DEGs between the low-CRRS and high-CRRS groups. Based on the results analyzed by the STRING database, the PPI network of DEGs was visualized by Cytoscape 3.6.1 software. DEGs with purple circles were considered upregulated in the high-CRRS group; nevertheless, those with bright blue circles were more enriched in the low-CRRS group (Fig. **9A**).

Subsequently, with the Cytohubba application, we finally obtained the top 10 hub genes from the network, namely, ACTN2, MYL2, LDB3, MYL1, MYL3, TNNI1, NEB, ACTC1, ACTA1, and MYH8. Single-gene analyses were then conducted to assess the effects of ACTN2 in HNSCC patients. Comparing the gene expression, the normal samples exhibited higher expression of ACTN2 than HNSCC samples (Fig. **9B**). However, according to the K‒M survival analysis, HNSCC patients with low ACTN2 expression had a better prognosis than those with high ACTN2 expression (Fig. **9C**). Additionally, there were no significant ACTN2 expression differences among the different clinicopathological features (Fig. **9D**). For assessments of TME scores, patients with higher expression of ACTN2 presented higher stromal scores as well as estimate scores (Fig. **9E**). However, as indicated by the CIBERSORT results, patients in the low-expression groups exhibited more CD8^+^ T-cell infiltration and less immune function activation (Fig. **9F** and **G**). The expression correlation analysis indicated that ACTN2 was positively correlated with most ICI-related genes. Moreover, concerning the comparison of IPS, patients with different ACTN2 expression exhibited similar immunotherapeutic responses to PD-1 and CTLA4 (Fig. **9H** and **I**).

## 4 DISCUSSION

HNSCC is the sixth most common malignancy globally, threatening human life and leading to poor prognosis. Given these findings, a novel signature for predicting prognosis and providing information regarding individualized therapy for HNSCC patients is necessary [3, 6, 10]. Chromatin regulators, according to previous studies, play important roles in tumor biological processes, including tumorigenesis and immune infiltration [16, 18]. Based on previous studies, CRs were considered potential biomarkers for prognosis and predicting the clinical therapeutic effects in tumors [17]. Nevertheless, the correlation between CRs and HNSCC requires further exploration. Herein, we identified different HNSCC subtypes and constructed a novel chromatin-related gene signature to explore their effects on prognostic prediction and immune infiltration. To our knowledge, this study is the first to use bioinformatic technology to explore the roles of CRs and establish a scoring system for HNSCC patients.

Referring to the expression of CRRGs, patients were divided into two clusters, which exhibited significant differences in prognosis, immune infiltration and immunotherapeutic response. As indicated by the results, patients in Cluster 2 exhibited an immunosuppressive TIME with less immune cell infiltration and lower immune scores, suggesting that Cluster 2 may gain fewer benefits from immunotherapy. Given these findings, patients can be distinguished clearly by different molecular subtypes and handled with different treatments. Similarly, we further established a CRRS system to assess the risk of patients and divided them into two risk groups (low-CRRS and high-CRRS) based on the prognostic DECRRGs. According to our analysis, this risk model can reliably predict the prognosis of HNSCC patients in both the training set and test set with satisfactory statistical results. Patients with different clinicopathological features can be clearly distinguished based on individual CRRSs. In addition, concerning the correlation analysis, an increasing CRRS decreased the immune score, immune cell infiltration, and immune function activation but increased the stromal score and TMB. These results from the correlation analysis suggested that HNSCC patients with lower CRRSs had more activation of immune-related processes and a better response to PD-1/CTLA4 inhibitor therapy; nevertheless, higher CRRSs may lead to mutation, metastasis, and immunosuppression. Nevertheless, patients in the high-CRRS group also exhibited higher expression of other ICI-related genes (*e.g.,* CD276), which may inform a novel immune treatment for immunosuppressive patients. Considering the above results, the information for HNSCC patients can be clearly predicted and assessed by our novel CRRS system, including prognosis, clinicopathological features, TMB, and TIME.

In this study, we also explored the effectiveness of nine model genes in HNSCC based on multiple landscape analyses. According to a previous study, FXR1 plays a crucial role in the progression of cyclin-dependent kinase inhibitors and p53, which can increase DNA damage and lead to cellular senescence [20, 21]. Overexpressed FXR1, as reported by Majumder *et al.,* can promote the destabilization of the p21 gene, reducing the expression of the p21 protein in oral tumors [20]. In addition, Qie *et al.* indicated that Fbxo4-mediated degradation of Fxr1 can effectively suppress tumorigenesis in HNSCC and that the increase in FXR1 can inhibit cellular senescence and dilute the response to tumor therapy [22]. Similar results were also obtained in our analysis, with an HR > 1 indicating that the increased expression of FXR1 may enhance the risk of HNSCC patients and promote a worse prognosis. Regarding the PRKAA2 gene, Jia *et al.* noted that this subunit of AMP-activated protein kinase influences the cellular energy production pathway and can be affected by overexpressed Trop2 in oral cancers [23]. Moreover, the decreased expression of USP49 can promote tumorigenesis and resistance to chemotherapeutic agents *via* the FKBP51-AKT pathway, and VRK1 may regulate the G1-S phase of the cell cycle [24, 25]. In addition, UTY exhibits several predictive abilities in HNSCC *via* noninvasive high-throughput multiplex ligation-dependent probe amplification technology, as determined by Sethi *et al.* [26]. Moreover, the remaining model genes were also mentioned and applied to establish the predictive signature according to previous studies [27, 28].

Similarly, we constructed the core gene network of DEGs between the low-CRRS and high-CCRRS groups. As suggested by the series analysis, ACTN2 had the highest score in the hub gene network and was expressed differently between the normal and HNSCC samples. Accordingly, the increased expression of ACTN2 enhanced the risk of HNSCC as well as immune cell infiltration (not including CD8^+^ T cells). Referring to the study by Shaikh *et al.,* ACTN2 was identified as an essential hub gene either in samples from tobacco-smoking or nonsmoking HNSCC patients by depicting muscle development-related biological progression, which coincided with our analysis of the hub genes [29].

An ideal and effective treatment for HNSCC is essential and remains to be explored. Recently, an individual treatment plan combining immunotherapy and chemotherapy for HNSCC has become prevalent and provides several satisfactory benefits. Due to immunosuppression and drug resistance, a precise assessment system for patients is necessary for predicting and evaluating the effectiveness of immuno-/chemotherapy and providing suitable and accurate guidelines. In our research, we predicted the effectiveness of immunotherapy and chemotherapy between the clusters and the CRRS groups to provide reliable advice for clinical treatment. Concerning the comparison of IPS in the two clusters, patients in Cluster 1 exhibited a higher IPS when treated with PD-1/CTLA4 inhibitors alone or in combination, which revealed that Cluster 1 was more sensitive to immunotherapy with PD-1 and CTLA4; nevertheless, the drug sensitivity analysis of the five chemotherapeutic agents determined that Cluster 2 had lower IC50 values for cisplatin, gemcitabine and methotrexate, indicating that Cluster 2 was more sensitive to chemotherapy. Similarly, while assessing clinical therapeutic effects based on the CRRS, patients with low CRRSs were more sensitive to immunotherapy, but patients with high CRRSs exhibited better chemotherapy effects. The correlation analysis also supported the results that a high CRRS decreased the expression of ICI genes and the IC50 values of chemotherapeutic agents. In addition, patients in Cluster 1 or the low-CRRS group appeared to have more enriched CD8^+^ T cells, which can influence the PD-1/PD-L1-related immune inhibitory axis to disrupt immune tolerance and kill cancer cells as well as strengthen the immunotherapy response [6, 30]. Therefore, while an HNSCC patient is considered a Cluster 1 subtype or is assessed with a low CRRS, immunotherapy mainly focused on PD-1/CTLA-4 blockade may result in a better prognosis. However, for Cluster 2 patients with high CRRSs, chemotherapeutic agents should provide more benefits. Considering the above results, patients diagnosed with HSNCC can be administered precise and individualized treatments based on assessment with our predictive risk model.

In summary, we divided HNSCC patients into two molecular subtypes in this study and constructed a novel CRRS signature for predicting prognosis, clinical correlation, TMB, TIME, immunotherapy, and chemotherapy. The results were reliable and may provide several guidelines for exploring more effective and satisfactory treatments for each patient. However, several limitations also appeared in our study. As a retrospective study, all of the above results were analyzed and determined based on a public database, which may cause potential selection bias. To decrease the bias, we applied an external cohort to test the predictive reliability of the risk model and multiple external databases and calculating technology to assess the TIME and clinical therapy. Therefore, these results can be considered reliable and stable. Similarly, as a predictive model, there is a lack of large sample cohorts to verify the predictive effects. Prospective studies with experimental assays and clinical information are necessary and crucial for further exploration and verification. Further studies should focus more on the relationship between experimental assays and clinical characteristics to investigate and confirm the results of our biomarkers with large samples.

## CONCLUSION

In this study, we identified molecular subtypes and established a novel predictive signature based on CRs. Referring to our analysis, this scoring system can possibly provide a novel research direction for exploring the correlation between CRs and HNSCC and requires further experimental validation.

## Data Availability

Data was openly obtained from the public database, including TCGA, GEO, TCIA, and TISCH databases, and available from the corresponding author upon request.

## LIST OF ABBREVIATIONS

CR: Chromatin Regulator
HNSCC: Head and Neck Squamous Cell Carcinoma
CRRG: CR-related Genes
K-M: Kaplan‒Meier
AUC: Areas Under the Curve
ROC: Receiver Operating Characteristic
OS: Overall Survival
ICI: Immune Checkpoint Inhibitor
TIME: Tumor Immune Microenvironment
TCGA: The Cancer Genome Atlas
TMB: Tumor Mutation Burden
GEO: Gene Expression Omnibus
PCA: Principal Component Analysis
GSVA: Gene Set Variation Analysis
ssGSEA: Single-sample Gene Set Enrichment Analysis
uni-Cox: Univariate Cox
IPS: Immunophenoscore
DECRRGs: Differentially Expressed CRRGs
logFC: log2 fold Change
LASSO: Least Absolute Shrinkage and Selection Operator
multi-Cox: Multivariate Cox
CRRS: Chromatin Regulator Risk Score
IC50: Half-maximum Inhibitory Concentration
DEGs: Differentially Expressed Genes
STRING: Search Tool for the Retrieval of Interacting Gene/Protein
PPI: Protein‒protein Interaction

## STANDARDS OF REPORTING

STROBE guidelines were followed in this study.
