# Supplementary material for: Development of Chromatin Regulator-related Molecular Subtypes and a Signature to Predict Prognosis and Immunotherapeutic Response in Head and Neck Squamous Cell Carcinoma
Source: Curr Cancer Drug Targets. 2024 Jan 4;24(8):804–19. doi: 10.2174/0115680096274798231121053634 (PMC11340294; doi:10.2174/0115680096274798231121053634)
Supplement: Supplementary file 1 [file CCDT-24-804_SD1.pdf]

Supplementary Materials

Development of Chromatin Regulator-related Molecular Subtypes and a Signature to Predict Prognosis and Immunotherapeutic Response in Head and Neck Squamous Cell Carcinoma

Juntao Huang[1,#,*], Ziqian Xu[2,#], Zhenzhen Wang[1], Chongchang Zhou[1] and Yi Shen[1,3,4,*]

[1]*Department of Otolaryngology-Head and Neck Surgery, Ningbo Medical Center Lihuili Hospital, The Affiliated Lihuili Hospital of Ningbo University, Ningbo, Zhejiang, China;* [2]*Department of Dermatology, Ningbo First Hospital, Ningbo, China;* [3]*Centre for Medical Research, Ningbo No.2 Hospital, Ningbo, China;* [4]*School of Medicine, Ningbo University, Ningbo, China*

THE LIST OF CHROMATIN-RELATED GENES BASED ON PREVIOUS RESEARCHES.

A1CF; ACTB; ACTL6A; ACTL6B; ACTR2; ACTR3; ACTR3B; ACTR5; ACTR6; ACTR8; ADNP; AEBP2; AHCTF1; AICDA; AIRE; AKAP1; ALKBH1; ALKBH3; ANP32A; ANP32B; ANP32E; APBB1; APEX1; APOBEC1; APOBEC2; APOBEC3A; APOBEC3B; APOBEC3C; APOBEC3D; APOBEC3F; APOBEC3G; APOBEC3H; ARID1A; ARID1B; ARID2; ARID3A; ARID3B; ARID3C; ARID4A; ARID4B; ARID5A; ARID5B; ARNTL; ARRB1; ASCL1; ASCL2; ASF1A; ASF1B; ASH1L; ASH2L; ASXL1; ASXL2; ASXL3; ATAD2; ATAD2B; ATF2; ATF7IP; ATM; ATN1; ATR; ATRX; ATXN7; ATXN7L3; AURKA; AURKB; AURKC; BABAM1; BAG6; BAHD1; BANF1; BANF2; BANP; BAP1; BARD1; BAZ1A; BAZ1B; BAZ2A; BAZ2B; BBX; BCL10; BCOR; BCORL1; BMI1; BOD1; BPTF; BRCA1; BRCA2; BRCC3; BRD1; BRD2; BRD3; BRD4; BRD7; BRD8; BRD9; BRDT; BRE; BRMS1; BRMS1L; BRPF1; BRPF3; BRWD1; BRWD3; BTAF1; BUB1; C14orf169; C17orf49; CARM1; CBX1; CBX2; CBX3; CBX4; CBX5; CBX6; CBX7; CBX8; CDC6; CDC73; CDK1; CDK17; CDK2; CDK3; CDK5; CDK7; CDK9; CDY1; CDY2A; CDYL; CDYL2; CECR2; CENPC; CHAF1A; CHAF1B; CHD1; CHD1L; CHD2; CHD3; CHD4; CHD5; CHD6; CHD7; CHD8; CHD9; CHEK1; CHMP1A; CHMP1B; CHRAC1; CHTOP; CHUK; CIC; CIR1; CIT; CLNS1A; CLOCK; CRB2; CREBBP; CSNK2A1; CSRP2BP; CTBP1; CTBP2; CTCF; CTCFL; CTR9; CUL1; CUL2; CUL3; CUL4A; CUL4B; CUL5; CXXC1; DAPK3; DAXX; DBF4; DBF4B; DDB1; DDB2; DDX21; DDX50; DEK; DHX30; DIDO1; DMAP1; DNAJC1; DNAJC2; DND1; DNMT1; DNMT3A; DNMT3B; DNMT3L; DNTT; DNTTIP2; DOT1L; DPF1; DPF2; DPF3; DPPA3; DPY30; DR1; DTX3L; DUSP1; DZIP3; E2F6; EED; EHMT1; EHMT2; EID1; EID2; EID2B; ELP2; ELP3; ELP4; ELP5; ELP6; EMSY; ENY2; EP300; EP400; EPC1; EPC2; ERBB4; ERCC6; ERCC6L; ERCC6L2; EXOSC1; EXOSC2; EXOSC3; EXOSC4; EXOSC5; EXOSC6; EXOSC7; EXOSC8; EXOSC9; EYA1; EYA2; EYA3; EYA4; EZH1; EZH2; FAM175A; FAM175B; FBL; FBRS; FBRSL1; FBXL19; FMR1; FOXA1; FOXO1; FOXP1; FOXP2; FOXP3; FOXP4; FTO; FXR1; FXR2; GABRG1; GADD45A; GADD45B; GADD45G; GATAD1; GATAD2A; GATAD2B; GFI1; GFI1B; GLYATL1; GLYR1; GSE1; GSG2; GTF2I; GTF3C1; GTF3C4; HAT1; HBP1; HCFC1; HCFC2; HDAC1; HDAC10; HDAC11; HDAC2; HDAC3; HDAC4; HDAC5; HDAC6; HDAC7; HDAC8; HDAC9; HDGF; HELLS; HIF1AN; HINFP; HIRA; HIRIP3; HJURP; HLCS; HLTF; HMG20A; HMG20B; HMGA1; HMGA2; HMGB1; HMGB2; HMGB3; HMGN1; HMGN2; HMGN3; HMGN4; HMGN5; HN1; HN1L; HNF1A; HNRNPA1; HP1BP3; HR; HSPA1A; HUWE1; IDH1; IDH2; IFIT3; IGFBP7; IKBKAP; IKZF1; IKZF3; ING1; ING2; ING3; ING4; ING5; INO80; INO80B; INO80C; INO80D; INO80E; JADE1; JADE2; JADE3; JAK2; JARID2; JDP2; JMJD1C; JMJD4; JMJD6; JMJD7; JMJD8; KANSL1; KANSL2; KANSL3; KAT2A; KAT2B; KAT5; KAT6A; KAT6B; KAT7; KAT8; KDM1A; KDM1B; KDM2A; KDM2B; KDM3A; KDM3B; KDM4A; KDM4B; KDM4C; KDM4D; KDM4E; KDM5A; KDM5B; KDM5C; KDM5D; KDM6A; KDM6B; KDM7A; KDM8; KEAP1; KMT2A; KMT2B; KMT2C; KMT2D; KMT2E; KMT5A; KMT5B; KMT5C; L3MBTL1; L3MBTL2; L3MBTL3; L3MBTL4; LAS1L; LBR; LEO1; LMNA; LMNB1; LMNB2; LRWD1; MAP3K7; MAPKAPK3; MASTL; MAX; MAZ; MBD1; MBD2; MBD3; MBD4; MBD5; MBD6; MBIP; MBTD1; MCRS1; MDC1; MDM2; MDM4; MEAF6; MECOM; MECP2; MEN1; MGA; MGEA5; MGMT; MIER1; MIER2; MIER3; MINA; MIS18BP1; MLLT1; MLLT10; MLLT3; MLLT6; MOCS1; MORF4; MORF4L1; MORF4L2; MOV10; MPHOSPH8; MRGBP; MSH6; MSL1; MSL2; MSL3; MST1; MTA1; MTA2; MTA3; MTF2; MUM1; MYBBP1A; MYO1C; MYSM1; NAA60; NAP1L1; NAP1L2; NAP1L3; NAP1L4; NAP1L5; NASP; NAT10; NBN; NCL; NCOA1; NCOA2; NCOA3; NCOA4; NCOA5; NCOA6; NCOA7; NCOR1; NCOR2; NEK6; NEK9; NFRKB; NFYB; NFYC; NIPBL; NOC2L; NPAS2; NPM1; NPM2; NSD1; NSL1; OGT; ORC1; ORC2; PADI1; PADI2; PADI3; PADI4; PAF1; PAGR1; PAK2; PARG; PARP1; PARP2; PARP3; PARP4; PATZ1; PAXIP1; PBK; PBRM1; PCGF1; PCGF2; PCGF3; PCGF5; PCGF6; PCNA; PDP1; PDS5A; PDS5B; PELP1; PES1; PHC1; PHC2; PHC3; PHF1; PHF10; PHF12; PHF13; PHF14; PHF19; PHF2; PHF20; PHF20L1; PHF21A; PHF21B; PHF23; PHF3; PHF6; PHF7; PHF8; PHIP; PIWIL4; PKM; PKN1; POGZ; POLE3; PPARGC1A; PPM1G; PPP2CA; PPP4C; PPP4R2; PPP4R3A; PPP4R3B; PPP4R3CP; PRC1; PRDM1; PRDM10; PRDM11;

1873-5576/24 © 2024 Bentham Science Publishers

PRDM12; PRDM13; PRDM14; PRDM15; PRDM16; PRDM2; PRDM4; PRDM5; PRDM6; PRDM7; PRDM8; PRDM9; PRKAA1; PRKAA2; PRKAB1; PRKAB2; PRKAG1; PRKAG2; PRKAG3; PRKCA; PRKCB; PRKCD; PRKDC; PRMT1; PRMT2; PRMT3; PRMT5; PRMT6; PRMT7; PRMT8; PRMT9; PRPF31; PRR12; PRR14; PSIP1; PWWP2B; PYGO1; PYGO2; RAC3; RAD51; RAD54B; RAD54L; RAD54L2; RAG1; RAG2; RAI1; RARA; RB1; RBBP4; RBBP5; RBBP7; RBP1; RBX1; RCC1; RCOR1; RCOR2; RCOR3; REST; REV1; RING1; RIT1; RLIM; RMI1; RNF168; RNF17; RNF2; RNF20; RNF40; RNF8; RPS6KA3; RPS6KA4; RPS6KA5; RRP8; RSAD1; RSF1; RTF1; RUVBL1; RUVBL2; RYBP; SAFB; SAP130; SAP18; SAP25; SAP30; SAP30L; SATB1; SATB2; SCMH1; SCML1; SCML2; SCML4; SENP1; SENP3; SET; SETBP1; SETD1A; SETD1B; SETD2; SETD3; SETD4; SETD5; SETD6; SETD7; SETD8P1; SETD9; SETDB1; SETDB2; SETMAR; SF3B1; SF3B3; SFMBT1; SFMBT2; SFPQ; SGF29; SHPRH; SIN3A; SIN3B; SIRT1; SIRT2; SIRT3; SIRT4; SIRT5; SIRT6; SIRT7; SKP1; SLF1; SMARCA1; SMARCA2; SMARCA4; SMARCA5; SMARCAD1; SMARCAL1; SMARCB1; SMARCC1; SMARCC2; SMARCD1; SMARCD2; SMARCD3; SMARCE1; SMC1A; SMCHD1; SMYD1; SMYD2; SMYD3; SMYD4; SMYD5; SNAI2; SND1; SP1; SP100; SP110; SP140; SPEN; SPOP; SRCAP; SRRM2; SRSF1; SRSF3; SS18L1; SS18L2; SSRP1; STK31; STK4; SUDS3; SUPT16H; SUPT3H; SUPT6H; SUPT7L; SUV39H1; SUV39H2; SUZ12; SYNCRIP; TADA1; TADA2A; TADA2B; TADA3; TAF1; TAF10; TAF12; TAF1L; TAF2; TAF3; TAF4; TAF5; TAF5L; TAF6; TAF6L; TAF7; TAF8; TAF9; TAF9B; TBL1XR1; TCF4; TDG; TDRD1; TDRD12; TDRD3; TDRD5; TDRD7; TDRD9; TDRKH; TERF1; TERF2; TET1; TET2; TET3; TEX10; TFDP1; TFF1; TFPT; TLE1; TLE2; TLE4; TLK1; TLK2; TNP1; TNP2; TONSL; TOP2A; TOP2B; TOPBP1; TOX; TOX2; TOX3; TOX4; TP53; TP53BP1; TRDMT1; TRIM16; TRIM24; TRIM27; TRIM28; TRIM33; TRRAP; TSPY1; TSPYL1; TSPYL2; TSPYL4; TSPYL5; TSPYL6; TSSK6; TTF2; TTK; TXN2; TYW5; UBE2A; UBE2B; UBE2D1; UBE2D3; UBE2E1; UBE2H; UBE2N; UBE2T; UBN1; UBR2; UBR5; UBR7; UBTF; UCHL5; UHRF1; UHRF2; UIMC1; UNK; USP11; USP12; USP15; USP16; USP17L2; USP21; USP22; USP3; USP36; USP44; USP46; USP49; USP7; UTY; VDR; VEZF1; VPS72; VRK1; WAC; WDR5; WDR77; WDR82; WHSC1; WHSC1L1; WSB2; XRCC1; YAF2; YEATS2; YEATS4; YWHAB; YWHAE; YWHAZ; YY1; ZBTB16; ZBTB24; ZBTB33; ZBTB38; ZBTB4; ZBTB7C; ZCWPW1; ZCWPW2; ZFAT; ZFP57; ZGPAT; ZHX1; ZHX2; ZHX3; ZMYM1; ZMYM2; ZMYM3; ZMYM4; ZMYM5; ZMYM6; ZMYND11; ZMYND8; ZNF217; ZNF516; ZNF532; ZNF541; ZNF592; ZNF687; ZNF711; ZNHIT1; ZRANB3; ZZZ3;
